# Sensitive and Selective Electrochemical Detection of Epirubicin as Anticancer Drug Based on Nickel Ferrite Decorated with Gold Nanoparticles

**DOI:** 10.3390/mi12111334

**Published:** 2021-10-30

**Authors:** Mohammad Mehmandoust, Nevin Erk, Ceren Karaman, Fatemeh Karimi, Sadegh Salmanpour

**Affiliations:** 1 Department of Analytical Chemistry, Faculty of Pharmacy, Ankara University, 06560 Ankara, Turkey; mehmandoust@ankara.edu.tr; 2 Biomaterials, Energy, Photocatalysis, Enzyme Technology, Nano & Advanced Materials, Additive Manufacturing, Environmental Applications, and Sustainability Research & Development Group (BIOENAMS R&D Group), Sakarya University, 54187 Sakarya, Turkey; 3 Department of Electricity and Energy, Vocational School of Technical Sciences, Akdeniz University, 07070 Antalya, Turkey; 4 Department of Chemical Engineering, Quchan University of Technology, Quchan 94771-67335, Iran; fkm024@qiet.ac.ir; 5 Department of Chemistry, Islamic Azad University, Sari Branch, Sari 194-48164, Iran; sadeghsalmanpour@gmail.com

**Keywords:** epirubicin, anticancer, monitoring, nickel ferrite, gold nanoparticles

## Abstract

The accurate and precise monitoring of epirubicin (EPR), one of the most widely used anticancer drugs, is significant for human and environmental health. In this context, we developed a highly sensitive electrochemical electrode for EPR detection based on nickel ferrite decorated with gold nanoparticles (Au@NiFe_2_O_4_) on the screen-printed electrode (SPE). Various spectral characteristic methods such as Fourier transform infrared spectra (FT-IR), X-ray diffraction (XRD), field emission scanning electron microscopy (FESEM), transmission electron microscopy (TEM), ultraviolet-visible spectroscopy (UV-Vis), energy-dispersive X-ray spectroscopy (EDX) and electrochemical impedance spectroscopy (EIS) were used to investigate the surface morphology and structure of the synthesized Au@NiFe_2_O_4_ nanocomposite. The novel decorated electrode exhibited a high electrocatalytic activity toward the electrooxidation of EPR, and a nanomolar limit of detection (5.3 nM) was estimated using differential pulse voltammetry (DPV) with linear concentration ranges from 0.01 to 0.7 and 0.7 to 3.6 µM. The stability, selectivity, repeatability reproducibility and reusability, with a very low electrode response detection limit, make it very appropriate for determining trace amounts of EPR in pharmaceutical and clinical preparations.

## 1. Introduction

**Scheme 1:**
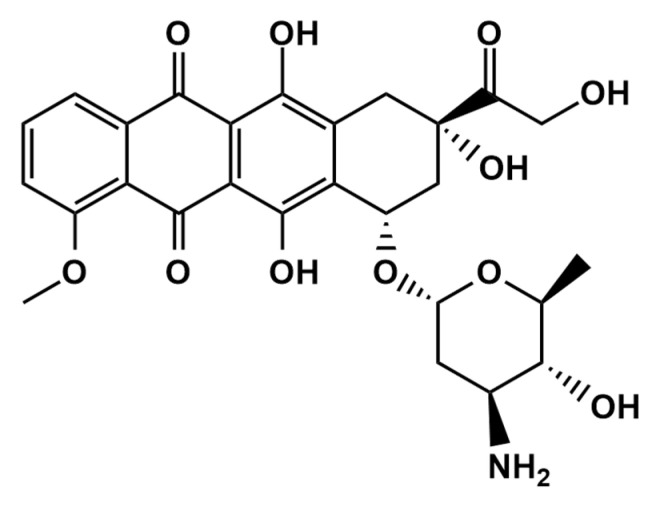
The chemical structure of epirubicin.

Epirubicin (EPR) (Scheme 1) is an anthracycline topoisomerase II inhibitor used for chemotherapy. Epirubicin, as an antitumor and anthracycline antibiotic derivative of doxorubicin, has been widely utilized for clinical therapy [1,2]. Doxorubicin and epirubicin(4′-epidoxorubicin) have only one difference, the spatial orientation of the 4′-moiety, and it has been exhibited effectively for treating leukemia, sarcoma and lymphoma [3,4]. EPR is an anticancer medication that works by selectively killing cancer cells rather than harming them by quickly dividing cells. Therefore, there may be a relationship between the EPR concentration and the clinical response. Low plasma EPR concentrations may suggest an ineffective prescription medication since the levels may be inadequate due to a significant molecular or complete cytotoxic response. As a result, measuring EPR levels in real biological samples is crucial for clinical diagnosis. In addition, cardiac problems such as heart failure and hair loss are crucial in the significant side effects of EPR and controlling the EPR concentration in the human body after cancer therapy.

EPR analysis has used a variety of analytical methods, including ultraviolet–visible spectroscopy (UV–Vis) [5,6], high-performance liquid chromatography (HPLC) [7,8,9,10,11], fluorimetry [12,13], mass spectrometry [14] and liquid chromatography–mass spectrometry/mass-spectrometry (LC-MS/MS) [15]. However, most of these techniques have drawbacks, such as long response times, complex analysis, high prices and limited sensitivity [16,17]. Voltammetric techniques, because of their quick reaction, high sensitivity and selectivity, and electrochemical techniques, particularly square wave voltammetry (SWV) and differential pulse voltammetry (DPV), have been used [18,19,20]. According to previous research, the performances of the electrochemical methods described are more appropriate than the other methods due to merit, such as a quick response to the analyte and the selectability in the presence of interfering agents [21,22,23,24,25,26]. Modifying electrode surfaces can remarkably enhance the surface sensitivity with appropriate stability and reproducibility [27,28,29,30,31]. Lately, the electrochemical properties of various binary metal oxides have been investigated widely due to their excellent conductivity, lower activation energy, appropriate structural stability and comparatively higher specific capacitance compared to the unitary metal oxides [32,33,34]. Nanomaterials have garnered a great deal of interest since they may be utilized in a variety of applications, including medical, electronics, energy storage and conversion systems, wastewater treatments, photovoltaic cells, catalysts, etc. [35,36,37,38,39]. Magnetic nanoparticles with the general formula MFe_2_O_4_ (M = Fe, Ni, Co, Cu, Mn) are among the most widely used materials in analytical chemistry, medicine and biotechnology. Due to their distinct benefits, they are increasingly being used for enzymes and other bioactive molecules. Nickel ferrite (NiFe_2_O_4_) nanoparticles (NPs) are a promising material for modified electrodes among various spinel transition metal oxides due to their high theoretical capacities (914 mA h/g), appropriate conductivity and low toxicity [40,41,42]. Due to its solid superparamagnetic characteristics, superior biocompatibility, high adsorbability and ease of production, NiFe_2_O_4_ nanoparticles are increasingly attracting interest in sensor and biosensor design [43]. Moreover, NiFe_2_O_4_ NPs show a high active surface area and low mass transfer resistance [44]. Metal nanoparticles (MNPs) are considered the most promising alternative for altering magnetic nanoparticle surfaces because of their size and their physical and chemical properties. The size and shape of metal nanoparticles are well recognized to impact electrochemical activity in sensing applications significantly. Due to their unique electrical, chemical and catalytic characteristics, various-shaped gold nanoparticles (Au NPs) such as nanospheres, nanorods and nanowires are widely utilized to construct electrochemical electrodes [45,46]. In addition, AuNPs have several benefits, including flexibility in size and shape management and excellent electrical and catalytic activity because of their superior conductivity and wide active surface area in the electrode [47,48]. These characteristics suggest that sensing applications aim to enhance the electron transport between the target molecules’ redox centers and electrode surfaces [49]. Firstly, the gold nanoparticles decorated on a nickel ferrite (Au@NiFe_2_O_4_) nanocomposite was synthesized in this work. There is no other original work on the electrochemical detection of EPR by Au@NiFe_2_O_4_ modified on SPE. Chemically produced Au@NiFe_2_O_4_ has a unique combination of characteristics, including a large electroactive surface area, good electron transferability and adsorptive properties, making it an appropriate sensing material for electrochemical determination EPR. The nanocomposite was characterized using field emission scanning electron microscopy (FESEM), transmission electron microscopy (TEM), electrochemical impedance spectroscopy (EIS), UV–Vis, Fourier transform infrared spectra (FT-IR) and energy-dispersive X-ray spectroscopy (EDX). The screen-printed electrode (SPE) modified using a Au@NiFe_2_O_4_ nanocomposite (Au@NiFe_2_O_4_/SPE) exhibited an outstanding detection limit of 5.3 nM and extremely selective electrode in the presence of interfering agents with two different concentration ranges, 0.01 to 0.7 and 0.7 to 3.6 µM. In addition, EPR analysis with satisfactory recovery in real samples (human plasma, injection and urine) was first performed using a Au@NiFe_2_O_4_/SPE. In comparison to the other approaches, it can be claimed that the analytical method used in this study is preferable.

## 2. Materials and Methods

### 2.1. Materials

Epirubicin was purchased from the Council of Europe (France); Nickel(II) chloride (NiCl_2_·6H_2_O), Ferric chloride(FeCl_3_ 6H_2_O), ethylene glycol, polyvinylpyrrolidone (PVP), Gold (III) chloride trihydrate (HAuCl4 3H_2_O, ≥49%), ascorbic acid, potassium hexacyanoferrate(III) and potassium hexacyanoferrate(II) were purchased from Sigma Aldrich Co (Burlington, MA, USA). Sera-Flex human blood plasma samples were also purchased from Dyna-Tek Industries Inc. (Lenexa, KS, USA). Additionally, a urine sample was obtained from a volunteer for use in the experiments. Epirubicin injection was also obtained from a local pharmacy. All of these substances were of analytical grade, and di-ionized water was utilized. The supporting electrolyte was 0.1 M Britton–Robinson buffer (B-R) prepared by mixing acetic acid (CH_3_COOH), phosphoric acid (H_3_PO_4_), boric acid (H_3_BO_3_) and potassium chloride (KCl) with deionized water. The pH of the supporting electrolyte was monitored with a pH meter (Hanna Instruments, Woonsocket, Rhode Island, USA). In addition, a stock EPR solution in the deionized water was prepared before each experiment.

### 2.2. Instrumentation

The electrochemical techniques such as differential pulse voltammetry (DPV), cyclic voltammetry (CV) and chronoamperometry (CA) were conducted using a Metrohm-Autolab potentiostat/galvanostat system (PGSTAT128N, Metrohm, Herisau, Sweden). Electrochemical impedance spectroscopy was performed under a 0.1 Hz to 100 kHz frequency using an IVIUM Compactstat (Eindhoven, The Netherlands) device. The employed screen-printed electrode had a Ag/AgCl paste and Pt electrodes on their own surface, as a reference and a counter electrode, respectively. UV–Vis was recorded using a double beam spectrophotometer (Shimadzu, Kyoto, Japan) model UV-1800 and quartz cells (Hellma, Müllheim, Germany). SEM and EDX were observed micrographs of the materials by ZEISS GeminiSEM 560 at 3.00 kV. The X-ray diffraction pattern was recorded using a Rigaku smart laboratory diffractometer (operated at 40 kV and 20 mA) with a Cu Kα source at a wavelength of 1.540 Å. TEM images were performed using an FEI Tecnai G2 Spirit microscope (Thermo Fisher Scientific, Waltham, MA, USA) at 120 kV. All electrochemical measurements were performed at 27.5 °C unless otherwise specified.

### 2.3. Synthesis of NiFe_2_O_4_

Firstly, 4.76 g of NiCl_2_·6H_2_O (0.02 mol) and 10.82 g of FeCl_3_·6H_2_O (0.04 mol) were dissolved in 30 mL of ethylene glycol (solution 1). Secondly, 5.0 g of urea and 0.4 g of polyvinylpyrrolidone (PVP) were dissolved in 30 mL of ethylene glycol (solution 2). Thirdly, solution 1 and solution 2 were mixed and stirred at 400 rpm at 30 min with a magnetic stirrer. Finally, this solution was moved to Teflon, a lined hydrothermal vessel, and heated at 180 °C for 20 h. The hydrothermal vessel was cooled at room temperature, and NiFe_2_O_4_ NPs were collected with a magnet. The NiFe_2_O_4_ NPs were washed with water (three times) and ethanol (two times) and dried at 70 °C for 12 h.

### 2.4. Synthesis of Au@NiFe_2_O_4_

Firstly, 70 mg of the powder obtained from magnetite nanoparticles was ultrasonicated in 20 mL of deionized water for 30 min to gain a uniform solution. Then, 700.0 µL of a HAuCl_4_ (0.1 g mL^−1^) solution was added to the reaction mixture at 70 °C. Afterward, after 10 min, 560 µL of ascorbic acid (0.5 g mL^−1^) was added to the reaction mixture, the stirring condition was maintained until the solution showed a slightly reddish color, and finally, the solution was cooled to room temperature and was separated using a magnet to form a magnetite-gold nanostructure. Next, Au@NiFe_2_O_4_ nanocomposites were washed four times with deionized water, and the resulting brown precipitate was dried at 70 °C.

### 2.5. Preparation of Au@NiFe_2_O_4_/SPE

The electrode surfaces were polished by 0.05-mm alumina slurries for 10 min and washed using a mixed solution of ethanol and water (1:1, *v*/*v*). Then, the mirror electrodes were dried at 25 °C for 1 h under argon gas. The electrode surfaces were modified using the optimized amount and concentration of NiFe_2_O_4_ and Au@NiFe_2_O_4_ nanocomposites (7.0 µL, 1.0 mg mL^−1^) on the screen-printed electrode surface. The solvent was removed using an infrared heat lamp. Then, the developed electrodes were obtained as a NiFe_2_O_4_/SPE and a Au@NiFe_2_O_4_/SPE. All electrodes were kept in a sealed box without fluctuations of temperature and pressure [50].

### 2.6. Preparation of Real Samples

Human plasma, urine and injections were used as real samples to detect the EPR content using the standard addition method. Additionally, the urine sample was obtained from candidates without a disease history. The urine samples were filtered thoroughly using PTFE (0.45-micrometer) membrane filters. A certain amount of EPR solution was added to the urine solution to prepare EPR spiked. To prepare the human plasma sample, the acquired plasma sample was kept at 20 °C until the test to produce the human plasma sample. Firstly, an aliquot of plasma was fortified with EPR to reach a concentration of 5.0 mM EPR. Then, 1.0 mL of plasma sample containing EPR was treated with 1.0 mL of acetonitrile, employed as a plasma protein precipitating agent. The precipitated proteins were separated by centrifugation for 15 min at 10,000 rpm after a 45-s vortex step. Finally, appropriate volumes of the supernatant were transferred to a volumetric flask and diluted with pH 4.0 B-R buffer to an acceptable volume. To prepare an injection, a certain number of injections were diluted with the B-R buffer solution and then investigated to measure EPR using DPV, analyzing without more purification.

## 3. Results

### 3.1. Characterizations of Au@NiFe_2_O_4_ Nanocomposite

**Figure 1:**
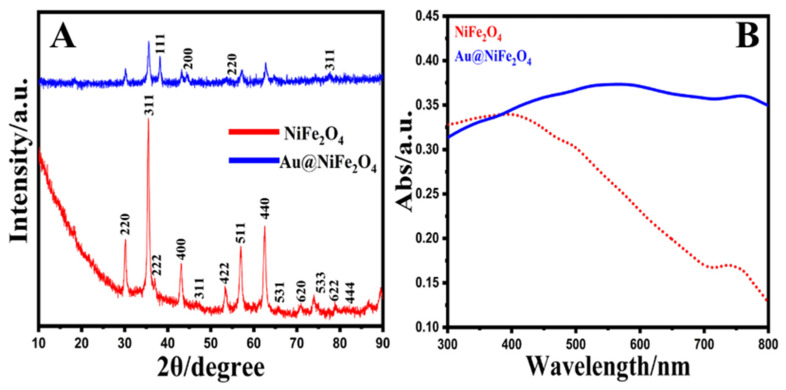
(**A**); XRD patterns of NiFe_2_O_4_ and Au@NiFe_2_O_4_ (**A**,**B**); UV–Vis spectra of NiFe_2_O_4_ and Au@NiFe_2_O_4_.

XRD patterns were utilized to illustrate the crystal structure, size and phase purity of the produced NiFe_2_O_4_ NPs and Au@NiFe_2_O_4_ nanocomposites (Figure 1A). The NPs were manually ground in an agate mortar for the powder XRD analysis sample. The planes (220), (311), (222), (400), (422), (511) and (440) corresponded to the 2θ values 30.5°, 35.6°, 38.1°, 43.4°, 54.2°, 57.6° and 63.2°. As a result, the sample has a face-centered cubic (FCC) structure and may be classified as an inverse spinel NiFe_2_O_4_ (ICCD 00-044-1458) [51]. The particle sizes of the nickel oxide and gold nanoparticles were investigated by Equation (1). The mean nanoparticle sizes were 32.6 and 14.0 nm for synthesized NiFe_2_O_4_ and Au nanoparticles, respectively. The particle size, *D*, was estimated using Scherrer’s equation, which is as follows [52]:

(1) $$D=\frac{k\lambda }{\beta cose\theta }$$

where *k* presents the shape coefficient for reciprocal lattice point (0.9), *β* shows the FWHM of the peak, *ϴ* exhibits the Bragg angle and *λ* presents the wavelength of X-rays = 1.54 Å. Further, the XRD of Au@NiFe_2_O_4_ exhibits four additional peaks located at 38.1°, 44.1°, 54.6° and 76.4°, which are attributed to the (111), (200), (220) and (311) planes of Au, respectively, with the JCPDS ref. No 89-3697 [53]. Thus, the XRD patterns affirm the deposition of Au nanoparticles on the surface of covalently grafted NiFe_2_O_4_.

The FT-IR spectra for the crystalline NiFe_2_O_4_ and Au@NiFe_2_O_4_ nanocomposites were observed in the region from 4000 to 450 cm^−1^, as exhibited in Figure S1. The band located at 1375 and 3444 cm^−1^ attributed to the O-H bond bending and stretching vibrations, respectively [54]. The two main metal-oxygen bands at 695 and 487 cm^−1^ are exhibited in the spectrum of the prepared NiFe_2_O_4_. These two bands are attributed to the vibration of ions in the crystal lattices, and the vibration absorption peak of C-O-Fe appears at 1115 cm^−1^. The 1317 cm^−1^ peak is assigned to the characteristic—CH_3_ bending [55]. In the case of Au@NiFe_2_O_4_, the intensity increased due to the doping of Au nanoparticles on NiFe_2_O_4_. The above evidence strongly proves the successful covalent binding of Au@NiFe_2_O_4_. Figure 1B exhibits the UV–Vis spectra of the pure NiFe_2_O_4_ and Au@NiFe_2_O_4_ nanocomposites. Only the side-band adsorption at 750 nm was found in the UV–Vis spectra of NiFe_2_O_4_, which can be attributed to the d–d transition from Ni3d-_t2g_ to Ni3d-_eg_ [56]. NiFe_2_O_4_ has a conventional spinel structure, with Ni^2+^ and Fe^3+^ occupying the tetrahedral and octahedral positions of the cubic spinel lattice, respectively [57]. At the same time, the spectra of the Au@NiFe_2_O_4_ were dominated by strong absorptions in 570 and 745 nm due to the presence of Au nanoparticles on the surface of NiFe_2_O_4_. These results reaffirmed the synthesis of the Au@NiFe_2_O_4_ nanocomposite.

**Figure 2:**
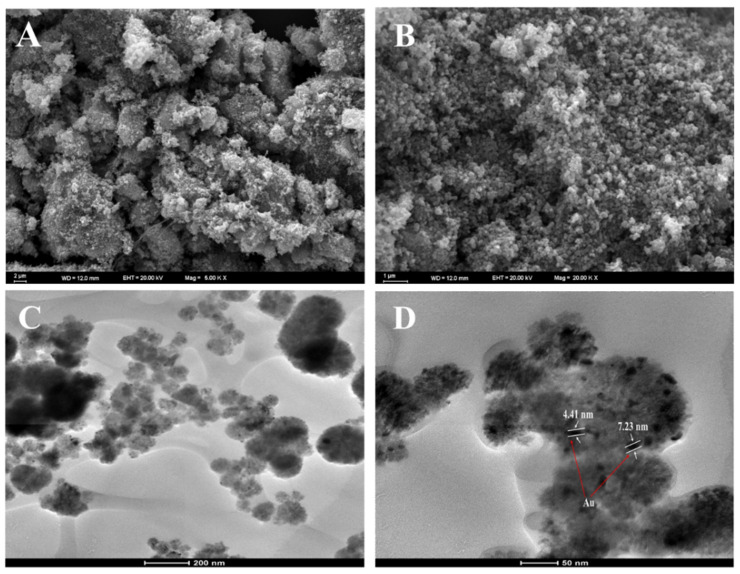
(**A**); SEM image of NiFe_2_O_4_, (**B**); SEM image of Au@NiFe_2_O_4_, (**C**,**D**); TEM image of Au nanoparticles deposited on the NiFe_2_O_4_.

The structure and morphology of the NiFe_2_O_4_ and Au@NiFe_2_O_4_ samples and their chemical composition are analyzed using SEM micrographs. Figure 2A,B present SEM images of NiFe_2_O_4_ nanoparticles and the Au@NiFe_2_O_4_ nanocomposite. There is a significant amount of aggregation of particles observed in the SEM image of NiFe_2_O_4_. Figure 2B shows that many of the Au NPs with a diameter of about 10.0 nm adhered to the surface of the NiFe_2_O_4_ nanocomposite. The results suggested that Au nanoparticles were immobilized on the NiFe_2_O_4_ nanocomposite.

Moreover, the TEM images of Au@NiFe_2_O_4_ exhibited in Figure 2C,D indicate the presence of AuNPs over the NiFe_2_O_4_ nanocomposite. Thus, the AuNP immobilized NiFe_2_O_4_ nanocomposite may increase the electron-transfer process by increasing the electrical conductivity to enhance the electrochemical detection potential. Energy-dispersive X-ray analysis was also utilized to analyze the elemental compositions of the nanocomposite and its synergistic components (Figure S2 and Table S1). The results demonstrate that the Au@NiFe_2_O_4_ nanocomposite was successfully manufactured. As a result, all the spectroscopic and microscopic studies of the nanocomposite’s production method and size are compelling.

### 3.2. Electrochemical Performance

#### 3.2.1. Influence of Modifier on the Electrochemical Oxidation of EPR

**Figure 3:**
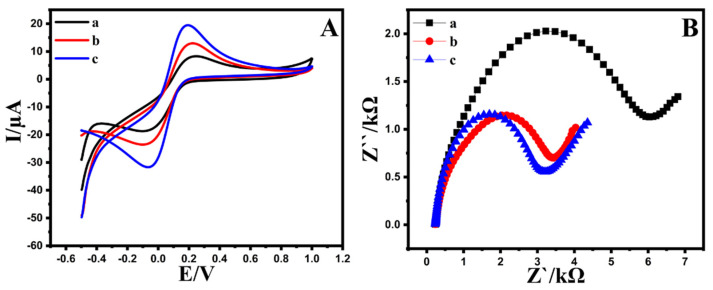
(**A**); CVs and (**B**); EIS at (a) bare SPE, (b) NiFe_2_O_4_/SPE, (c) Au@NiFe_2_O_4_/SPE in the presence of 5.0 mM [Fe(CN)_6_]^3−/4−^ as redox probe containing 0.1 M KCl.

Cyclic voltammetry and DPV voltammograms were utilized to assess the redox behavior of the Au@NiFe_2_O_4_/SPE in a 0.1 M B-R buffer as a supporting electrolyte and compared with the NiFe_2_O_4_/SPE and a bare electrode. Figure S3 shows DPVs recorded with different modified and bare electrodes. After the modified Au@NiFe_2_O_4_, the peak current of the 0.5 µM EPR was approximately 3.1 times higher than the bare electrode, indicating that the effect of the increased active surface area and conductivity by synergic affects the nanocomposite. Therefore, the Au@NiFe_2_O_4_/SPE was utilized for further experiments. Moreover, the cyclic voltammograms of bare and modified electrodes in the absence of EPR in 0.1 M B-R (pH 4.0) were obtained to evaluate the effect of the nanohybrid on the electroanalytical performance of the bare electrode. The findings demonstrated that in the absence of EPR, thanks to the large electroactive surface area and the synergistic effects between the Au and Ni Fe_2_O_4_ nanoparticles, the response of the Au@NiFe_2_O_4_/SPE was detected higher than that of the bare electrode (Figure S4). The electrochemical performance of the proposed electrochemical sensor was studied by determining the electron transfer rate using the solution as a redox probe molecule in 0.1 M KCl. For this purpose, the peak-to-peak distance (ΔE_p_) value between the anodic and cathodic signals, an essential indicator for the electron transfer rate, was determined at a bare SPE, the NiFe_2_O_4_/SPE and the Au@NiFe_2_O_4_/SPE with a scan rate of 50 mV s^−1^. As seen in Figure 3A, the ΔE_p_ value of the Au@NiFe_2_O_4_/SPE (ΔE_p_ = 239 mV) was lower than that of the bare electrode (ΔE_p_ = 301 mV), exhibiting the presence of a faster electron transfer. In addition, an improvement obtained at both the anodic and cathodic signals has clearly revealed the strong electrocatalytic activity of the Au@NiFe_2_O_4_. Furthermore, Figure S5A exhibits the CV voltammograms of the Au@NiFe_2_O_4_/SPE in the presence of 5.0 mM [Fe(CN)_6_]^3−/4−^ containing 0.1 M KCl at various scan rates from 10.0 to 300.0 mV s^−1^ and the plot of the peak current against the square root of scan rate is exhibited in Figure S5B. Figure S5A shows that with an increasing scan rate, the width of the voltammograms gradually increased. The anodic/cathodic peaks current density enhanced dramatically at high scan rates. Therefore, electron exchange happens slowly at the electrode surface to record peak currents at low scan rates, resulting in a thinner voltammograms with shorter current density peaks. The scan rate study revealed a clear relationship between the peak current and the specified range of the square roots of scan rates.

The electrochemical active surface area (EASA) of the Au@NiFe_2_O_4_/SPE was conducted using CV analysis in 0.1 M B-R buffer as a supporting electrolyte, containing 5.0 mM [Fe(CN)_6_]^3−/4−^ as a redox probe and compared with the bare electrode and the NiFe_2_O_4_/SPE. The EASA of the electrodes was evaluated using the slope value of the peak current against various square root scan rate plots with the Randles–Sevcik equation (Equation (S1)). As a result, the active surface area was observed at 0.26, 0.16 and 0.069 cm^2^ for the Au@NiFe_2_O_4_/SPE, the NiFe_2_O_4_/SPE and the bare SPE, respectively. On the other hand, the active surface area of the Au@NiFe_2_O_4_/SPE is about 3.8-fold higher than the area of the bare electrode, showing that the Au@NiFe_2_O_4_/SPE is the most appropriate for electrocatalytic sensing applications.

The electron transport characteristics of changed electrodes can also be studied using electrochemical impedance spectroscopy. The charge transfer resistance (R_ct_) for the electrodes could be observed through the semicircle diameter. Figure 3B exhibits the Nyquist plot of the Au@NiFe_2_O_4_/SPE, the NiFe_2_O_4_/SPE and the bare SPE in the presence of 5.0 mM [Fe(CN)_6_]^3−/4−^ in 0.1 M KCl. The R_ct_ at the bare SPE, the NiFe_2_O_4_/SPE and the Au@NiFe_2_O_4_/SPE are approximately 6.32, 4.33 and 2.97 kΩ, respectively. The semicircle diameter of the Au@NiFe_2_O_4_/SPE showing that Rct was decreased at the developed electrodes. The EIS measurements demonstrate that the Au@NiFe_2_O_4_/SPE has a better electrochemical activity and conductivity than the bare electrode. Therefore, it can be inferred that the Au@NiFe_2_O_4_/SPE indicates a synergistic effect by combining the Au and NiFe_2_O_4_ and the Au@NiFe_2_O_4_/SPE nanocomposite improves the electron transfer process.

#### 3.2.2. Heterogeneous Electron Transfer Rate Constant (K°)

The ability of the Au@NiFe_2_O_4_/SPE was investigated in increasing the rate of electron transfer. The electrical conductivity and surface resistance have a reciprocal relationship. The electrical conductivity of the Au@NiFe_2_O_4_/SPE is higher than the bare SPE and the NiFe_2_O_4_/SPE, suggesting improved electron transport kinetics. It is due to the greater surface area and higher conductivity of the Au@NiFe_2_O_4_/SPE. Equation (S2) is utilized to observe the standard heterogeneous rate constant. The K° values for the Au@NiFe_2_O_4_/SPE, the NiFe_2_O_4_/SPE and the bare electrode are 1.96 × 10^−8^, 1.22 × 10^−8^ and 8.41 × 10^−9^ cm s^−1^, respectively. The K° values represent the estimated kinetic facilities of redox couples. A higher K° achieves equilibrium in a shorter amount of time, implying a quicker electron transfer.

### 3.3. Optimization of Conditions for Developing Sensitive and Selective Au@NiFe_2_O_4_/SPE

#### 3.3.1. Effect of Physical and Chemical Properties

Optimal conditions affect the activity and sensitivity of the electrochemical electrode and can be useful for the sensitivity of the developed electrodes. Therefore, the following parameters were obtained: certain concentration and amount of nanocomposite, supporting electrolyte and its pH, scan rate, temperature and stirring of electrolyte at the Au@NiFe_2_O_4_/SPE.

To obtain the optimum concentration to achieve the highest current efficiency, the impact of the Au@NiFe_2_O_4_ dosage on the surface of the electrode was investigated, in the range of 0.1–2.0 mg mL^−1^, and the obtained data are exhibited in Figure S6. As shown, raising the Au@NiFe_2_O_4_ amount from 0.1 to 1.0 mg mL^−1^ increased the current percentage substantially. It might be because a greater surface area and more adsorption functional sites are available. On the other hand, at higher concentrations (higher than 1.0 mg mL^−1^), there is a significant decrease, which might be related to overlying or aggregating accessible binding sites and reducing the total available adsorbent surface area. In the next step, investigating the effect of the nanocomposite amount 4.0–10.0 µL of Au@NiFe_2_O_4_ suspension was dropped to the surface of the electrode, drying at room temperature. The voltammetric behavior of different Au@NiFe_2_O_4_ suspension amounts was studied using 5.0 µM EPR (Figure S7). The results showed that the highest I_p_ was obtained with 7.0 µL of Au@NiFe_2_O_4_. Upon a further increment in the amount of the suspension, the adherence of the modifier layer on the surface of the electrode would be reduced.

Furthermore, the analyte diffusion through the dense layer of modifier would be hindered, which causes a noticeable decrease in the sensitivity of the modified SPE. As a result, the optimal volume of Au@NiFe_2_O_4_ as a modifier nanocomposite, increasing the conductivity and activity to increase the electron transfer process, is 7.0 μL. The effect of various supporting electrolytes, various temperatures and stirring rates was also observed using DPV in the presence of 1.0 µM EPR at pH 4.0 (Figures S8–S10).

#### 3.3.2. The Effect of pH

**Figure 4:**
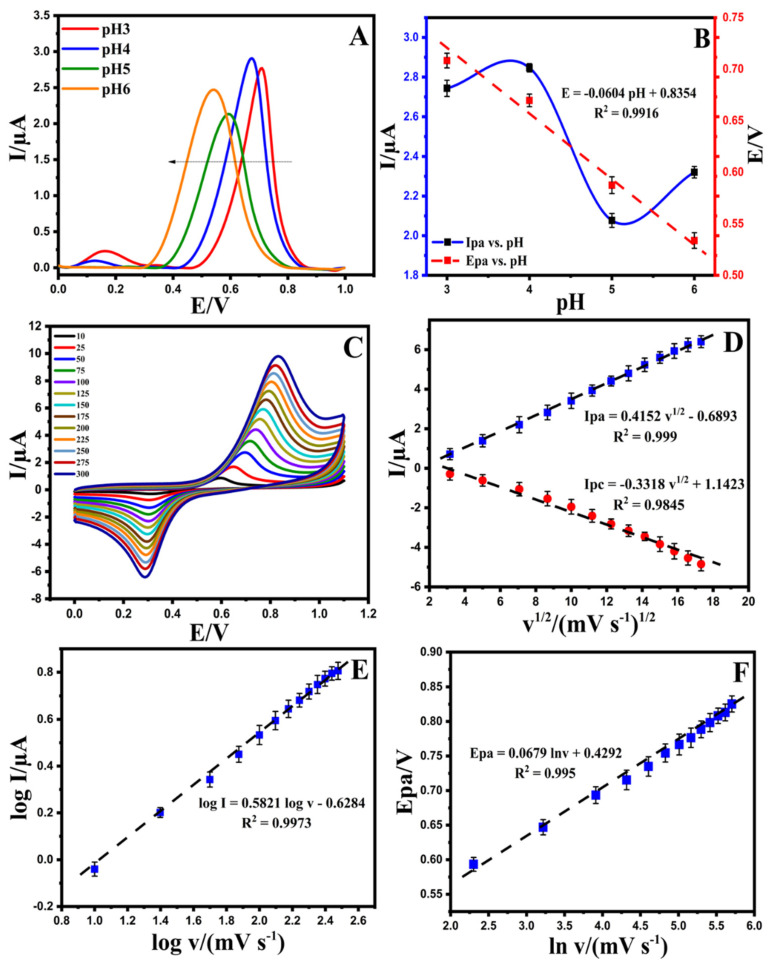
(**A**); Differential pulse voltammetry solution containing 5.0 µM EPR in the pH range of 3.0–6.0 at the Au@NiFe_2_O_4_/SPE, (**B**); Effect of pH values on the peak current (blue line) and peak potential (red line) of EPR on the surface of the Au@NiFe_2_O_4_/SPE at various pH values (from 2.0 to 8.0), (**C**); Cyclic voltammograms solution containing 5.0 µM EPR in the B-R buffer at pH 4.0 at diverse scan rate (10–300 mV s^−1^), (**D**); The plot of I_(pa,pc)_ vs. ν^1/2^ for 5.0 µM EPR on the surface of the Au@NiFe_2_O_4_/SPE, (**E**); The plot of the logarithm of the anodic peak current (log I_pa_) vs. the logarithm of scan rate (log v) obtained at the Au@NiFe_2_O_4_/SPE for 5.0 µM EPR in B-R buffer at pH 4.0, (**F**); The plot of E_pa_ vs. natural logarithm of scan rate (ln v) for EPR.

The pH is an essential factor in the electrochemical behavior of a developed electrode. The activity of EPR in real samples due to the pH of the solution can affect the adsorbent surface in the presence of OH^−^ and H^+^. According to the DPV signals of the Au@NiFe_2_O_4_/SPE in the range of pH 3.0–6.0, the oxidation current of EPR enhanced with an increase in the pH value from 3.0 to 4.0, and after pH 4.0, the signals decreased at the surface of the Au@NiFe_2_O_4_/SPE. Additionally, the oxidation peak potential of EPR shifted to the left by increasing the pH value (Figure 4A) with a slope of 60.4 mV/pH at the surface of the Au@NiFe_2_O_4_/SPE that is close to the Nernstian value (59.0 mV/pH) for an equal number of electrons and protons in the redox system (Figure 4B). The results confirm that the best oxidation current occurs at pH = 4.0, and this value is selected as the optimum pH for further experiments.

#### 3.3.3. The Effect of Scan Rate

**Scheme 2:**
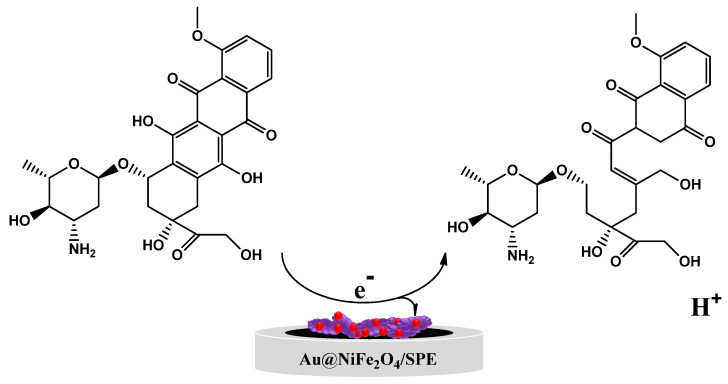
The mechanism of possible oxidation of EPR.

Figure 4C exhibits the effect of the scan rate on the redox peak current responses of the Au@NiFe_2_O_4_/SPE in the presence of 5.0 µM EPR containing a 0.1 M B-R buffer at a pH 4.0 scan rate ranging from 10.0 to 300.0 mV s^−1^. A systematic increase in I_pa_ and I_pc_ and ∆Ep was observed with an increase in the scan rate. The results of the relationship between the current of EPR and ν^1/2^ on the surface of the Au@NiFe_2_O_4_/SPE are shown in Figure 4D. The equations I_pa_ = 0.4152 ν^1/2^ −0.6893 (R^2^ = 0.999) and I_pc_ = −0.331 ν^1/2^ +1.1423 (R^2^ = 0.9845) were observed for this study for the oxidation and reduction in EPR. These equations and the linear relations between current and ν^1/2^ at the Au@NiFe_2_O_4_/SPE suggest that the electro-oxidation of EPR is under diffusion control. The slope of the logarithm of peak current vs. the logarithm of scan rates (Figure 4E) also reaffirmed that the electrode progression was a diffusion-controlled electrode process because the slop of the log I_p_ vs. log v is close to 0.5. Figure 4F exhibits a linear relationship between E_pa_ and ln v for EPR, as presented in the following equation: E_pa_ (V) = 0.0679 ln v + 0.4292 (R^2^ = 0.995). In Laviron’s theory, for a reversible electrode reaction, E_pa_ is determined by Equation (S3). Hence, the number of transferred electrons in the electro-oxidation of EPR is estimated to be 0.78 (≅1). The results confirmed that one electron and one proton are transferred during the electro-oxidation of EPR on the Au@NiFe_2_O_4_/SPE. Based on our observations using Laviron’s theory and slope of E_pa_ vs. pH, a possible electrode reaction mechanism proposed for EPR was illustrated in Scheme 2, in which one proton and one electron transfer was involved and caused the oxidation of the hydroxyl group to quinine. The results also demonstrated that the reversible electron process led to the formation of the quinolinic structure of EPR.

The corresponding results to optimize the modified electrode are shown in the supporting information. The following optimal experimental conditions were observed: the certain concentration of nanocomposite: 1.0 mg mL^−1^; the amount of nanocomposite: 7.0 μL; the optimal supporting electrolyte and pH value: B-R buffer at pH 4.0; the electrolyte temperature and stirring rate: 25.0 °C and 400 rpm.

#### 3.3.4. Chronoamperometric Study

**Figure 5:**
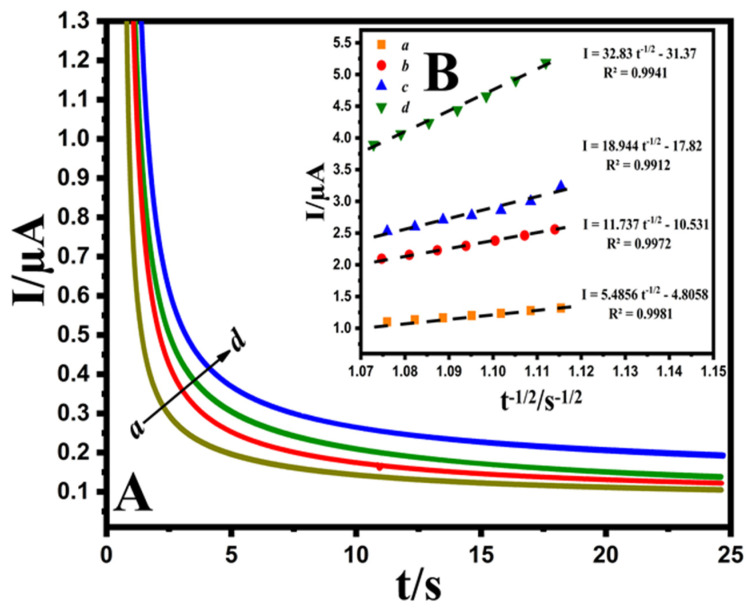
(**A**); Chronoamperograms obtained at the Au@NiFe_2_O_4_/SPE in the presence of (a) 200.0, (b) 300.0, (c) 400.0 and (d) 500.0 μM EPR at pH 4.0 B-R buffer. (**B**); Plots of I vs. t^−1/2^ derived from chronoamperograms.

The chronoamperometric evaluation was performed via adjusting the Au@NiFe_2_O_4_/SPE potentials at 0.6 V with different concentrations of EPR (200.0, 300.0, 400.0 and 500.0 μM) containing 0.1 M B-R buffer at pH 4.0 (Figure 5A). Cottrell’s equation (Equation (S4)) was utilized to describe the current responses (I) for the diffusion coefficient electrocatalytic procedures of electroactive substances. By plotting I versus t^−1/2^, the linear curve was observed for various concentrations of EPR. Afterward, the obtained direct lines’ slope was drawn against the EPR concentration (Figure 5B). Finally, the diffusion coefficient of 4.72 × 10^−6^ cm^2^ s^−1^ was evaluated for a developed EPR electrode.

### 3.4. Determination of EPR at the Au@NiFe_2_O_4_/SPE

**Figure 6:**
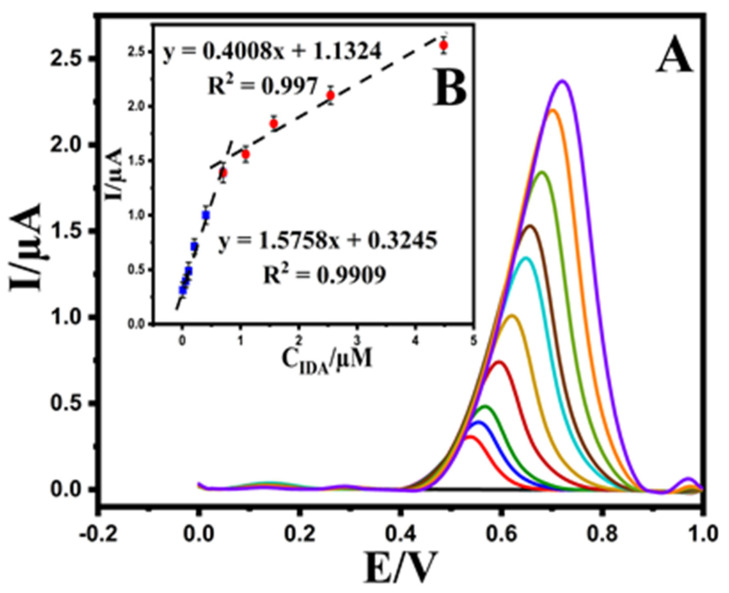
(**A**); Differential pulse voltammograms of EPR with increasing concentrations at the Au@NiFe_2_O_4_/SPE, (**B**); the plot of EPR concentrations versus oxidation current peak the Au@NiFe_2_O_4_/SPE in the concentration range of 0.01–3.6 μM.

The analytical performance of the Au@NiFe_2_O_4_/SPE was performed by analyzing EPR at diverse concentrations using DPV under optimized experimental conditions. As shown in Figure 6A, the oxidation peak current of EPR has enhanced linearly with increasing EPR concentrations at the ranges 0.01–0.7 and 0.7–3.6 μM with slopes of 1.5758 and 0.4008 μA μmol L^−1^, respectively. The corresponding linear regression equations were observed as I_pa_ (μA) = 1.5758 C_EPR_ (μM) + 0.3245 (R² = 0.9909) and I_pa_ = 0.4008C_EPR_ + 1.1324 (R^2^ = 0.997) (Figure 6B). The second linear segment’s reduction in sensitivity (slope) is most likely due to a kinetic limitation. Therefore, the limit of detection (LOD) of the Au@NiFe_2_O_4_/SPE was calculated as 5.32 nM (RSD = 5%) using the equation LOD = 3.3 s/m [22], where ‘s’ is the standard deviation of the 10 repeated measurements at the calibration range’s lowest concentration, and ‘m’ is the calibration curve’s slope, respectively. The analytical detection figures of merit are summarized in Table 1. These results suggest that the Au@NiFe_2_O_4_/SPE could be used in a real environment to detect medically relevant concentrations of EPR.

The obtained analytical parameters were compared to similar reported analytical sensors and other analytical methods such as HPLC, fluorescence and LC-MS/MS, which have been utilized to sense EPR (Table 2). The analytical performance of the Au@NiFe_2_O_4_/SPE, which has wide dynamic linearity and low LOD value, was almost much more appropriate than other comparative analytical methods toward detecting EPR.

### 3.5. Selectivity of Au@NiFe_2_O_4_/SPE

**Figure 7:**
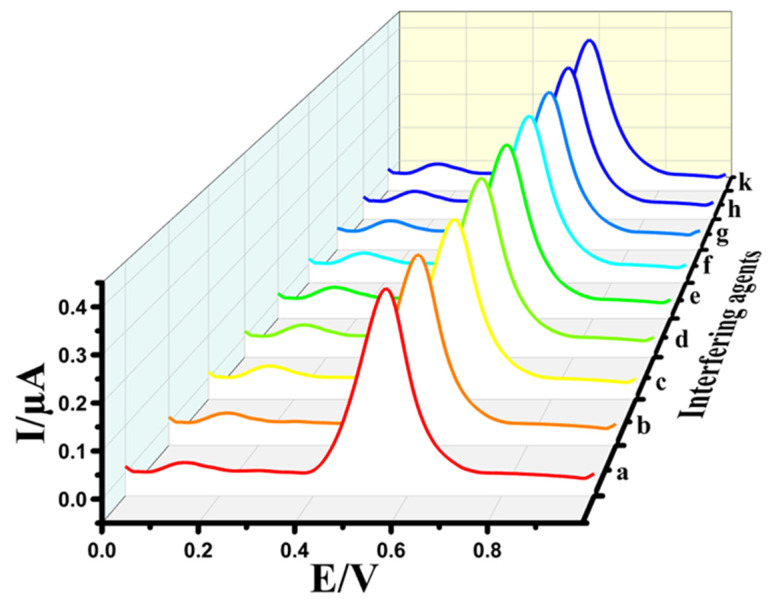
DPVs of 0.5 μM EPR (a) in the presence of various 200-fold ascorbic acid (b), uric acid (c), glucose (d), L-cysteine (e), L-arginine (f), dopamine (g), vitamin D (h) and vitamin B12 (k) at the Au@NiFe_2_O_4_/SPE.

The interference study is an essential factor for electrodes, which significantly impacts practical applications. To assess the selectivity of the Au@NiFe_2_O_4_/SPE, various concentrations of interfering agents such as ascorbic acid (b), uric acid (c), glucose (d), L-cysteine (e), L-arginine (f), dopamine (g), vitamin D (h) and vitamin B_12_ (k) were separately added into a 0.1 M B-R buffer at pH 4.0 containing 0.5 μM EPR (a). The results exhibited that a 200-fold excess of interfering agents did not show any or negligible interference effect in the determination of EPR (Figure 7). The corresponding relative errors for EPR were lower than ±5%, which correlates with the tolerance limit defined in the selectivity measurements, indicating that the Au@NiFe_2_O_4_/SPE has a promising selectivity for the determination of EPR. Therefore, the results suggest that the developed electrode has outstanding anti-interference activity and can be performed to EPR determination in biological samples.

### 3.6. Reproducibility, Repeatability, Stability and Reusability of the Au@NiFe_2_O_4_/SPE

**Figure 8:**
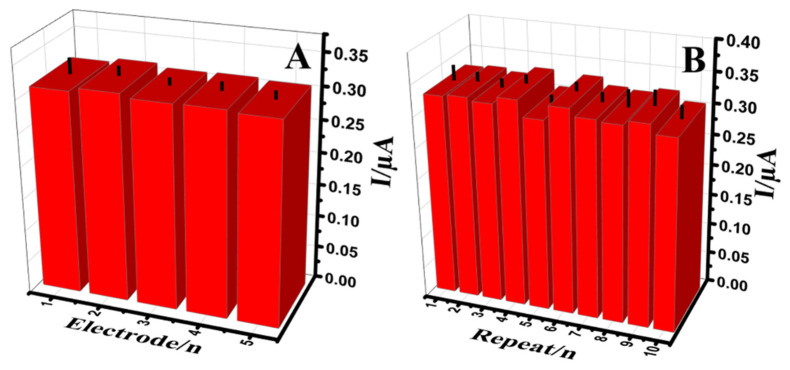
The histogram (**A**); Reproducibility (**B**); repeatability measurements of the Au@NiFe_2_O_4_/SPE in the presence of 0.5 µM EPR containing 0.1 M B-R buffer at pH 4.0.

The response of Au@NiFe_2_O_4_/SPE in a B-R buffer containing 0.5 μM of EPR was observed to study reproducibility (Figure 8A). The five individual electrodes were prepared at similar conditions, and the relative standard deviations (RSDs) less than 2.61% were obtained for EPR. The RSD of 2.1% for ten successive signals was observed confirming good repeatability for the Au@NiFe_2_O_4_/SPE as an electroanalytical electrode (Figure 8B). Moreover, to evaluate the longer-term response stability, two electrodes were kept in 0.1 M B-R (pH 4.0) at 4 °C. The developed electrodes were kept in glass vials with the tops wrapped using parafilm. After each week, two electrodes were utilized to use continuous voltammetry to measure the 0.5 µM EPR mixed with a 0.1 M B-R buffer (pH 4.0) (Figure S11). The observed signal showed 97.9% of its initial response relative to 0.5 µM EPR, using the Au@NiFe_2_O_4_/SPE, indicating the good stability, repeatability and reproducibility of the developed electrode. Finally, the reusability of the Au@NiFe_2_O_4_/SPE was observed. The Au@NiFe_2_O_4_/SPE is not a disposable electrode. It can be utilized at least 13 times by rinsing with 0.1 M B-R buffer (pH 4.0). Thanks to the strong covalent bond interaction between the uniformly dispersed Au nanoparticles and NiFe_2_O_4_ surface (notably, the Au[Fe–O] bond), the specific capacity and the long-term cyclic stability of the fabricated Au@NiFe_2_O_4_ nanohybrid was assured.

### 3.7. Real Samples Analysis

The Au@NiFe_2_O_4_/SPE was used to determine EPR in urine, injection and human plasma samples. To assess the target analytes in these real samples, the standard addition method was performed. According to the results obtained in Table 3, the developed electrode can be used directly to determine EPR in real samples. The relative recovery was utilized to estimate the accuracy of the results. The recoveries varied between 97.5 and 101.0% for human plasma, 98.4 and 103% for a urine sample and 102.6 and 105.2% for an injection with acceptable RSDs for a novel developed electrode. Table 3 confirms the high-performance ability of the Au@NiFe_2_O_4_/SPE as an EPR electrochemical electrode in the real samples.

## 4. Conclusions

This paper reports on a new EPR sensor design that consists of gold nanoparticles immobilized on the bimetallic nanocomposite on the surface of an SPE. The developed Au@NiFe_2_O_4_/SPE exhibits good electroactivity because of its high surface area and conductivity. A dynamic linearity range and detection limit of 0.01 to 0.7 and 0.7 to 3.6 μM and 5.3 nM were observed for the Au@NiFe_2_O_4_/SPE, respectively. The developed electrochemical sensor exhibits outstanding selectivity, linearity, repeatability, reproducibility, sensitivity and reusability in target detection. According to the recovery experiments and standard addition method, it could be said that EPR in real samples such as human plasma, urine and an injection did not affect the selective analysis of the anticancer drug. On the other hand, the applicability of the Au@NiFe_2_O_4_/SPE to the rapid analysis of EPR in real samples demonstrates the excellent ability for practical application. The developed sensor could be an outstanding candidate as an alternative analytical technique for determining the trace amount of EPR in clinical samples.

## Figures and Tables

**Table 1:** Determination figures of merit for EPR at Au@NiFe_2_O_4_/SPE.

Metrics	Au@NiFe_2_O_4_/SPE
response variability (RSD, %)	3.56
sensitivity (μA μmol L^−1^)	1.5758 ± 0.14 ^a^
linear dynamic range (μM)	0.01–0.7 and 0.7–3.6
correlation coefficient	0.9909 and 0.997
limit of detection (LOD, nM)	5.32

^a^ Mean ± Standard deviation for *n* = 3.

**Table 2:** Detection figures of merit for other methods for EPR reported in the literature.

Method	Modified Electrode	LOD (μM)	LWR ^a^ (μM)	Ref.
DPV ^b^	Ce-ZnO/GCE ^c^	0.0023	0.01–600	[58]
SWV ^d^	Au/MWNTs-ZnO/SPE	0.0025	0.005–0.2	[59]
LSV ^e^	SWNT–DCP/SPE ^f^	0.02	0.05–10	[60]
SWV	CoFe_2_O_4_/1,3-DPIBr ^g^/CPE	0.01	0.04–450	[61]
fluorescence	CMC-CdTe/ZnS QDs	0.04	-	[62]
HPLC	-	0.0149	1.87–187	[11]
LC–MS/MS	-	1.87	5.6–374.5	[15]
DPV	Au@NiFe_2_O_4_/SPE	0.0053	0.01–3.6	Our work

^a^ Linear working range, ^b^ differential pulse voltammetry, ^c^ cerium-doped ZnO nanoflowers, ^d^ square wave voltammetry, ^e^ linear sweep voltammetry, ^f^ single-walled carbon nanotube/dicetyl phosphate, ^g^ 1,3-dipropylimidazolium bromide.

**Table 3:** Analysis of EPR in real samples using Au@NiFe_2_O_4_/SPE.

Sample	Spiked (µM)	Found (µM) ^a^	RSD (%)	Recovery (%)
Human plasma	0.4	0.39 ± 0.01	2.14	97.5
	0.6	0.6 ± 0.05	2.84	100.1
	0.8	0.79 ± 0.04	3.52	98.8
	1.0	1.0 ± 0.01	2.98	100.0
Urine	0.4	0.41 ± 0.01	3.15	102.5
	0.6	0.59 ± 0.06	2.89	98.4
	0.8	0.80 ± 0.03	4.11	100.0
	1.0	1.03 ± 0.03	2.59	103.0
Injection	-	1.15 ± 0.14	-	-
	0.4	1.51 ± 0.08	3.7	102.6
	0.6	1.78 ± 0.09	4.27	105.2

^a^ All samples were analyzed using the standard addition method.

## Supplementary Materials

The following are available online at https://www.mdpi.com/article/10.3390/mi12111334/s1, Figure S1: FTIR spectra of the NiFe_2_O_4_ and Au@NiFe_2_O_4_ nanocomposites, Figure S2: EDX analysis of the Au@NiFe_2_O_4_ nanocomposite, Figure S3: DPVs of the Au@NiFe_2_O_4_/SPE, the NiFe_2_O_4_/SPE and the bare electrode in the presence of 0.5 µM EPR containing a 0.1 M BR buffer, Figure S4: The CVs of the bare electrode, the NiFe_2_O_4_/SPE and the Au@NiFe_2_O_4_/SPE in the absence of EPR in B-R at pH 4.0, Figure S5: (**A**) CVs of the Au@NiFe_2_O_4_/SPE in the presence of 5.0 mM [Fe(CN)6]3−/4− containing 0.1 M KCl at various scan rates from 10 to 400 mV/s, (**B**) The plot of the corresponding peak current against the square root of the scan rate., Figure S6: Effect of Au@NiFe_2_O_4_ concentration on EPR response using the Au@NiFe_2_O_4_/SPE with 5.0 µM EPR in the presence of 0.1 BR buffer at pH 4.0, Figure S7: Effect of Au@NiFe_2_O_4_ amount on EPR response using the Au@NiFe_2_O_4_/SPE with 5.0 µM EPR in the presence of 0.1 BR buffer at pH 4.0, Figure S8: Effect of the Au@NiFe_2_O_4_/SPE in the presence of 1.0 µM EPR with various supporting electrolytes, Figure S9: Effect of solution temperature on EPR response using the Au@NiFe_2_O_4_/SPE and the DPV method with 1.0 µM EPR in the presence of a 0.1 BR buffer at pH 4.0, Figure S10: Effect of solution stirring rates on EPR response using the Au@NiFe_2_O_4_/SPE and the DPV method with 1.0 µM EPR in the presence of a 0.1 BR buffer at pH 4.0, Figure S11: Long-term response stability of the Au@NiFe_2_O_4_ electrode exposed to 0.5 µM EPR over five weeks, Table S1: Percentage of elements present in the Au@NiFe_2_O_4_ nanocomposite.

## References

[1] Eksborg S., Hardell L., Bengtsson N.O., Sjodin M., Elfsson B. (1992). Epirubicin as a Single Agent Therapy for the Treatment of Breast-Cancer—A Pharmacokinetic and Clinical-Study. Med. Oncol. Tumor Pharmacother..

[2] Munster P., Marchion D., Bicaku E., Schmitt M., Lee J.H., DeConti R., Simon G., Fishman M., Minton S., Garrett C. (2007). Phase I trial of histone deacetylase inhibition by valproic acid followed by the topoisomerase II inhibitor epirubicin in advanced solid tumors: A clinical and translational study. J. Clin. Oncol..

[3] Lehmann J., Retz M., Weining C., Albers P., Frohneberg D.F., Becker T., Funke P.J., Walz P., Langbein S., Schiller M. (2003). Adjuvant systemic chemotherapy with cisplatin plus methotrexate (CM) versus methotrexate, vinblastine, epirubicin, and cisplatin (MVEC) for locally advanced bladder cancer: Results of a randomized, multicenter phase III study in Germany. J. Urol..

[4] Kuroda M., Kotake T., Akaza H., Hinotsu S., Kakizoe T., Grp, The Japanese Urothelial Cancer Research Group (1998). Efficacy of dose-intensified MEC (methotrexate, epirubicin and cisplatin) chemotherapy for advanced urothelial carcinoma: A prospective randomized trial comparing MEC and M-VAC (methotrexate, vinblastine, doxorubicin and cisplatin). Jpn. J. Clin. Oncol..

[5] Hajian R., Ekhlasi E., Daneshvar R. (2012). Spectroscopic and Electrochemical Studies on the Interaction of Epirubicin with Fish Sperm DNA. E-J. Chem..

[6] Charak S., Jangir D.K., Tyagi G., Mehrotra R. (2011). Interaction studies of Epirubicin with DNA using spectroscopic techniques. J. Mol. Struct..

[7] Dodde W.I., Maring J.G., Hendriks G., Wachters F.M., Groen H.J., de Vries E.G., Uges D.R. (2003). Determination of epirubicin and its metabolite epirubicinol in saliva and plasma by HPLC. Ther. Drug Monit..

[8] Fogli S., Danesi R., Innocenti F., Di Paolo A., Bocci G., Barbara C., Del Tacca M. (1999). An improved HPLC method for therapeutic drug monitoring of daunorubicin, idarubicin, doxorubicin, epirubicin, and their 13-dihydro metabolites in human plasma. Ther. Drug Monit..

[9] Gopinath P., Veluswami S., Thangarajan R., Gopisetty G. (2018). RP-HPLC-UV Method for Estimation of Fluorouracil-Epirubicin-Cyclophosphamide and Their Metabolite Mixtures in Human Plasma (Matrix). J. Chromatogr. Sci..

[10] Camaggi C.M., Comparsi R., Strocchi E., Testoni F., Pannuti F. (1988). Hplc Analysis of Doxorubicin, Epirubicin and Fluorescent Metabolites in Biological-Fluids. Cancer Chemother. Pharmacol..

[11] Tariq M., Thomas S., Singh A., Talegaonkar S. (2018). Developed and validated stability indicating HPLC method for the determination of epirubicin in bulk drug, marketed injection and polymeric nanoparticles. Braz. J. Pharm. Sci..

[12] Duffy P.M., Hayes M.C., Cooper A., Smart C.J. (1996). Determination and reversal of resistance to epirubicin intravesical chemotherapy. A flow cytometric model. Br. J. Urol..

[13] El-Kimary E.I., El-Yazbi A.F. (2016). An eco-friendly stability-indicating spectrofluorimetric method for the determination of two anticancer stereoisomer drugs in their pharmaceutical preparations following micellar enhancement: Application to kinetic degradation studies. Spectrochim. Acta A.

[14] Greco F., Arif I., Botting R., Fante C., Quintieri L., Clementi C., Schiavon O., Pasut G. (2013). Polysialic acid as a drug carrier: Evaluation of a new polysialic acid-epirubicin conjugate and its comparison against established drug carriers. Polym. Chem..

[15] Sottani C., Leoni E., Porro B., Montagna B., Amatu A., Sottotetti F., Quaretti P., Poggi G., Minoia C. (2009). Validation of an LC-MS/MS method for the determination of epirubicin in human serum of patients undergoing Drug Eluting Microsphere-Transarterial Chemoembolization (DEM-TACE). J. Chromatogr. B..

[16] Fotoohi K., Skarby T., Soderhall S., Peterson C., Albertioni F. (2005). Interference of 7-hydroxymethotrexate with the determination of methotrexate in plasma samples from children with acute lymphoblastic leukemia employing routine clinical assays. J. Chromatogr. B..

[17] Liu X.J., Liu J.Z., Huang Y.Y., Zhao R., Liu G.Q., Chen Y. (2009). Determination of methotrexate in human serum by high-performance liquid chromatography combined with pseudo template molecularly iniprinted polymer. J. Chromatogr. A.

[18] Wang F., Wu Y.J., Liu J.X., Ye B.X. (2009). DNA Langmuir-Blodgett modified glassy carbon electrode as voltammetric sensor for determinate of methotrexate. Electrochim. Acta.

[19] Gao L., Wu Y.J., Liu J.X., Ye B.X. (2007). Anodic voltammetric behaviors of methotrexate at a glassy carbon electrode and its determination in spiked human urine. J. Electroanal. Chem..

[20] Guo Y.J., Chen Y.H., Zhao Q., Shuang S.M., Dong C. (2011). Electrochemical Sensor for Ultrasensitive Determination of Doxorubicin and Methotrexate Based on Cyclodextrin-Graphene Hybrid Nanosheets. Electroanalysis.

[21] Karimi-Maleh H., Karimi F., Fu L., Sanati A.L., Alizadeh M., Karaman C., Orooji Y. (2022). Cyanazine herbicide monitoring as a hazardous substance by a DNA nanostructure biosensor. J. Hazard. Mater..

[22] Karimi-Maleh H., Yola M.L., Atar N., Orooji Y., Karimi F., Kumar P.S., Rouhi J., Baghayeri M. (2021). A novel detection method for organophosphorus insecticide fenamiphos: Molecularly imprinted electrochemical sensor based on core-shell Co_3_O_4_@MOF-74 nanocomposite. J. Colloid Interface Sci..

[23] Karimi-Maleh H., Orooji Y., Karimi F., Alizadeh M., Baghayeri M., Rouhi J., Tajik S., Beitollahi H.D., Agarwal S., Gupta V.K. (2021). A critical review on the use of potentiometric based biosensors for biomarkers detection. Biosens. Bioelectron..

[24] Karimi-Maleh H., Alizadeh M., Orooji Y., Karimi F., Baghayeri M., Rouhi J., Tajik S., Beitollahi H., Agarwal S., Gupta V.K. (2021). Guanine-Based DNA Biosensor Amplified with Pt/SWCNTs Nanocomposite as Analytical Tool for Nanomolar Determination of Daunorubicin as an Anticancer Drug: A Docking/Experimental Investigation. Ind. Eng. Chem. Res..

[25] Medetalibeyoglu H., Beytur M., Manap S., Karaman C., Kardas F., Akyildirim O., Kotan G., Yuksek H., Atar N., Yola M.L. (2020). Molecular Imprinted Sensor Including Au Nanoparticles/Polyoxometalate/Two-Dimensional Hexagonal Boron Nitride Nanocomposite for Diazinon Recognition. ECS J. Solid State Sci. Technol..

[26] Boke C.P., Karaman O., Medetalibeyoglu H., Karaman C., Atar N., Yola M.L. (2020). A new approach for electrochemical detection of organochlorine compound lindane: Development of molecular imprinting polymer with polyoxometalate/carbon nitride nanotubes composite and validation. Microchem. J..

[27] Karimi-Maleh H., Keyvanfard M., Alizad K., Fouladgar M., Beitollahi H., Mokhtari A., Gholami-Orimi F. (2011). Voltammetric Determination of N-Actylcysteine Using Modified Multiwall Carbon Nanotubes Paste Electrode. Int. J. Electrochem. Sci..

[28] Ensafi A.A., Dadkhah-Tehrani S., Karimi-Maleh H. (2011). A Voltammetric Sensor for the Simultaneous Determination of L-Cysteine and Tryptophan Using a p-Aminophenol-Multiwall Carbon Nanotube Paste Electrode. Anal. Sci..

[29] Ensafi A.A., Karimi-Maleh H., Mallakpour S. (2011). N-(3,4-Dihydroxyphenethyl)-3,5-dinitrobenzamide-Modified Multiwall Carbon Nanotubes Paste Electrode as a Novel Sensor for Simultaneous Determination of Penicillamine, Uric acid, and Tryptophan. Electroanalysis.

[30] Raoof J.B., Ojani R., Karimi-Maleh H. (2009). Electrocatalytic oxidation of glutathione at carbon paste electrode modified with 2,7-bis (ferrocenyl ethyl) fluoren-9-one: Application as a voltammetric sensor. J. Appl. Electrochem..

[31] Karaman C., Karaman O., Yola B.B., Ulker İ., Atar N., Yola M.L. (2021). A novel electrochemical Aflatoxin B1 immunosensor based on gold nanoparticles decorated porous graphene nanoribbon and Ag nanocubes incorporated MoS2 nanosheets. New J. Chem..

[32] Ozcan N., Karaman C., Atar N., Karaman O., Yola M.L. (2020). A Novel Molecularly Imprinting Biosensor Including Graphene Quantum Dots/Multi-Walled Carbon Nanotubes Composite for Interleukin-6 Detection and Electrochemical Biosensor Validation. ECS J. Solid State Sci. Technol..

[33] Huang L., Chen D.C., Ding Y., Feng S., Wang Z.L., Liu M.L. (2013). Nickel-Cobalt Hydroxide Nanosheets Coated on NiCo_2_O_4_ Nanowires Grown on Carbon Fiber Paper for High-Performance Pseudocapacitors. Nano Lett..

[34] Yang W.L., Gao Z., Ma J., Zhang X.M., Wang J., Liu J.Y. (2014). Hierarchical NiCo_2_O_4_@NiO core-shell hetero-structured nanowire arrays on carbon cloth for a high-performance flexible all-solid-state electrochemical capacitor. J. Mater. Chem. A.

[35] Al Sharabati M., Abokwiek R., Al-Othman A., Tawalbeh M., Karaman C., Orooji Y., Karimi F. (2021). Biodegradable polymers and their nano-composites for the removal of endocrine-disrupting chemicals (EDCs) from wastewater: A review. Environ. Res..

[36] Karimi F., Ayati A., Tanhaei B., Sanati A.L., Afshar S., Kardan A., Dabirifar Z., Karaman C. (2022). Removal of metal ions using a new magnetic chitosan nano-bio-adsorbent; A powerful approach in water treatment. Environ. Res..

[37] Karaman O., Ozdogan H., Uncu V.A., Karaman C., Tamar A.G. (2020). Investigation of the effects of different composite materials on neutron contamination caused by medical LINAC. Kerntechnik.

[38] Akca A., Karaman O., Karaman C., Atar N., Yola M.L. (2021). A comparative study of CO catalytic oxidation on the single vacancy and di-vacancy graphene supported single-atom iridium catalysts: A DFT analysis. Surf. Interfaces.

[39] Karimi-Maleh H., Ayati A., Davoodi R., Tanhaei B., Karimi F., Malekmohammadi S., Orooji Y., Fu L., Sillanpaa M. (2021). Recent advances in using of chitosan-based adsorbents for removal of pharmaceutical contaminants: A review. J. Clean. Prod..

[40] Wang Z., Zhang X., Li Y., Liu Z.T., Hao Z.P. (2013). Synthesis of graphene-NiFe_2_O_4_ nanocomposites and their electrochemical capacitive behavior. J. Mater. Chem. A.

[41] Venkatachalam V., Jayavel R. (2015). Novel Synthesis of Ni-Ferrite (NiFe_2_O_4_) Electrode Material for Supercapacitor Applications. AIP Conf. Proc..

[42] Anwar S., Muthu K.S., Ganesh V., Lakshminarasimhan N. (2011). A Comparative Study of Electrochemical Capacitive Behavior of NiFe_2_O_4_ Synthesized by Different Routes. J. Electrochem. Soc..

[43] Ensafi A.A., Jafari-Asl M., Rezaei B., Allafchian A.R. (2013). Simultaneous determination of guanine and adenine in DNA based on NiFe_2_O_4_ magnetic nanoparticles decorated MWCNTs as a novel electrochemical sensor using adsorptive stripping voltammetry. Sens. Actuators B Chem..

[44] Chen H., Yan J.Q., Wu H., Zhang Y.X., Liu S.Z. (2016). One-pot fabrication of NiFe_2_O_4_ nanoparticles on alpha-Ni(OH)(2) nanosheet for enhanced water oxidation. J. Power Sources.

[45] Tarkistani M.A.M., Komalla V., Kayser V. (2021). Recent Advances in the Use of Iron-Gold Hybrid Nanoparticles for Biomedical Applications. Nanomaterials.

[46] Dawson K., Baudequin M., O’Riordan A. (2011). Single on-chip gold nanowires for electrochemical biosensing of glucose. Analyst.

[47] Han Y.J., Zhang R., Dong C., Cheng F.Q., Guo Y.J. (2019). Sensitive electrochemical sensor for nitrite ions based on rose-like AuNPs/MoS2/graphene composite. Biosens. Bioelectron..

[48] Er E., Erk N. (2020). Construction of a sensitive electrochemical sensor based on 1T-MoS2 nanosheets decorated with shape-controlled gold nanostructures for the voltammetric determination of doxorubicin. Microchim. Acta.

[49] Prakash S., Chakrabarty T., Singh A.K., Shahi V.K. (2013). Polymer thin films embedded with metal nanoparticles for electrochemical biosensors applications. Biosens. Bioelectron..

[50] Yola M.L., Atar N. (2019). Development of cardiac troponin-I biosensor based on boron nitride quantum dots including molecularly imprinted polymer. Biosens. Bioelectron..

[51] Yang B., Wang C., Xiao R., Yu H.Y., Huang C.Q., Wang J.X., Xu J.L., Liu H.M., Xia F., Xiao J.Z. (2019). High NH3 selectivity of NiFe_2_O_4_ sensing electrode for potentiometric sensor at elevated temperature. Anal. Chim. Acta.

[52] Karaman C., Karaman O., Atar N., Yola M.L. (2021). Sustainable electrode material for high-energy supercapacitor: Biomass-derived graphene-like porous carbon with three-dimensional hierarchically ordered ion highways. Phys. Chem. Chem. Phys..

[53] Pawar R.C., Kang S., Ahn S.H., Lee C.S. (2015). Gold nanoparticle modified graphitic carbon nitride/multi-walled carbon nanotube (g-C3N4/CNTs/Au) hybrid photocatalysts for effective water splitting and degradation. RSC Adv..

[54] Amulya M.A.S., Nagaswarupa H.P., Kumar M.R.A., Ravikumar C.R., Prashantha S.C., Kusuma K.B. (2020). Sonochemical synthesis of NiFe_2_O_4_ nanoparticles: Characterization and their photocatalytic and electrochemical applications. Appl. Surf. Sci. Adv..

[55] Naidu T.M., Narayana P.L. (2019). Synthesis and Characterization of Fe-TiO_2_ and NiFe_2_O_4_ Nanoparticles and Its Thermal Properties. J. Nanosci. Technol..

[56] Deeth R.J., Anastasi A., Diedrich C., Randell K. (2009). Molecular modelling for transition metal complexes: Dealing with d-electron effects. Coord. Chem. Rev..

[57] Ceylan A., Ozcan S., Ni C., Shah S.I. (2008). Solid state reaction synthesis of NiFe_2_O_4_ nanoparticles. J. Magn. Magn. Mater..

[58] Jandaghi N., Jahani S., Foroughi M.M., Kazemipour M., Ansari M. (2020). Cerium-doped flower-shaped ZnO nano-crystallites as a sensing component for simultaneous electrochemical determination of epirubicin and methotrexate. Microchim. Acta.

[59] Wang Y.T., Xie J.M., Tao L., Tian H., Wang S., Ding H. (2014). Simultaneous electrochemical determination of epirubicin and methotrexate in human blood using a disposable electrode modified with nano-Au/MWNTs-ZnO composites. Sens. Actuators B Chem..

[60] Zhang H.J. (2004). Fabrication of a single-walled carbon nanotube-modified glassy carbon electrode and its application in the electrochemical determination of epirubicin. J. Nanoparticle Res..

[61] Karimi F., Shojaei A.F., Tabatabaeian K., Shakeri S. (2017). CoFe_2_O_4_ nanoparticle/ionic liquid modified carbon paste electrode as an amplified sensor for epirubicin analysis as an anticancer drug. J. Mol. Liq..

[62] Bardajee G.R., Sharifi M., Mahmoodian H. (2021). Novel CMC-CdTe/ZnS QDs Nanosensor for the Detection of Anticancer Drug Epirubicin. J. Fluoresc..

