# Supplementary material for: Sensitive and Selective Electrochemical Detection of Epirubicin as Anticancer Drug Based on Nickel Ferrite Decorated with Gold Nanoparticles
Source: Micromachines (Basel). 2021 Oct 30;12(11):1334. doi: 10.3390/mi12111334 (PMC8624078; doi:10.3390/mi12111334)
Supplement: Supplementary file 1 [file micromachines-12-01334-s001.zip › micromachines-1437444-supplementary.pdf]

Supplementary Materials: Sensitive and Selective Electrochemical Detection of Epirubicin as Anticancer drug Based on Nickel Ferrite Decorated with Gold Nanoparticles

Mohammad Mehmandoust, Nevin Erk, Ceren Karaman, Fatemeh Karimi

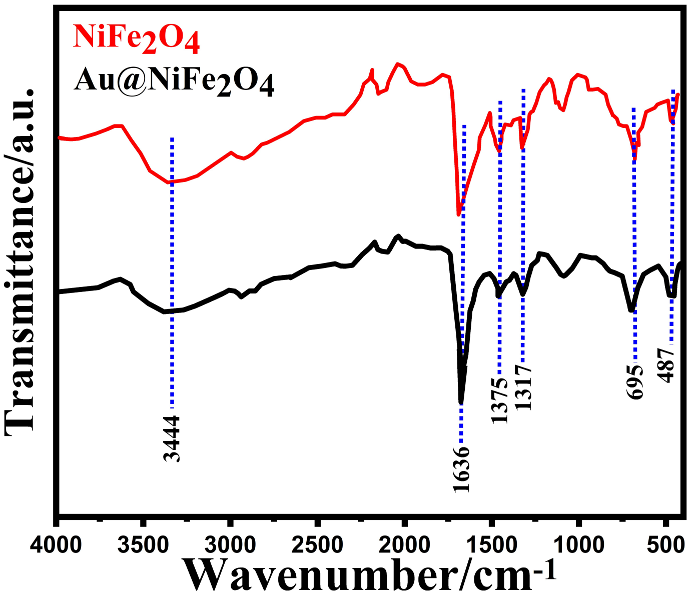

Figure S1. FTIR spectra of the NiFe2O4 and Au@NiFe2O4 nanocomposites.

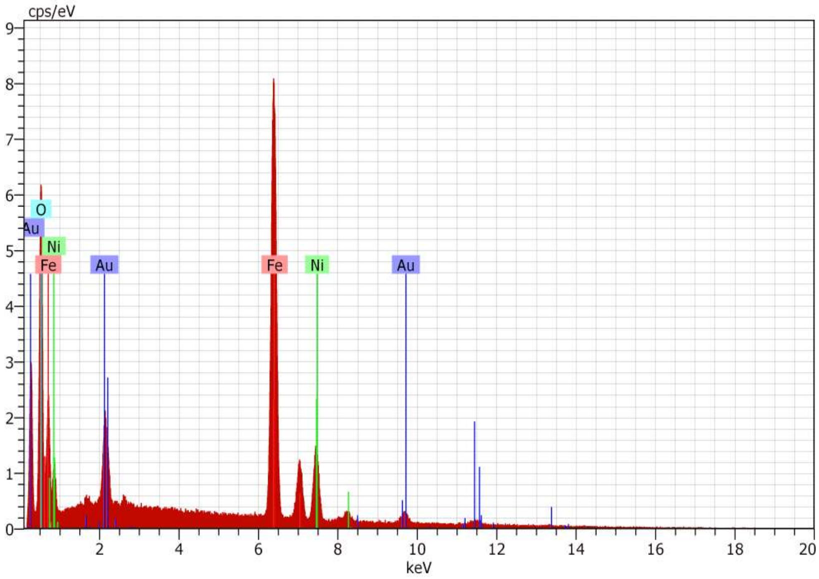

Figure S2. EDX analysis of the Au@NiFe2O4 nanocomposite.

Table S1. Percentage of elements present in the Au@NiFe2O4 nanocomposite.

El	Series	norm. C (wt.%)	Atom. C (at.%)	Error (%)
Fe	K-series	51.15	36.59	1.3
O	K-series	20.73	51.76	2.4
Au	L-series	15.69	3.18	0.5
Ni	K-series	12.43	8.46	0.4
Total		100	100	

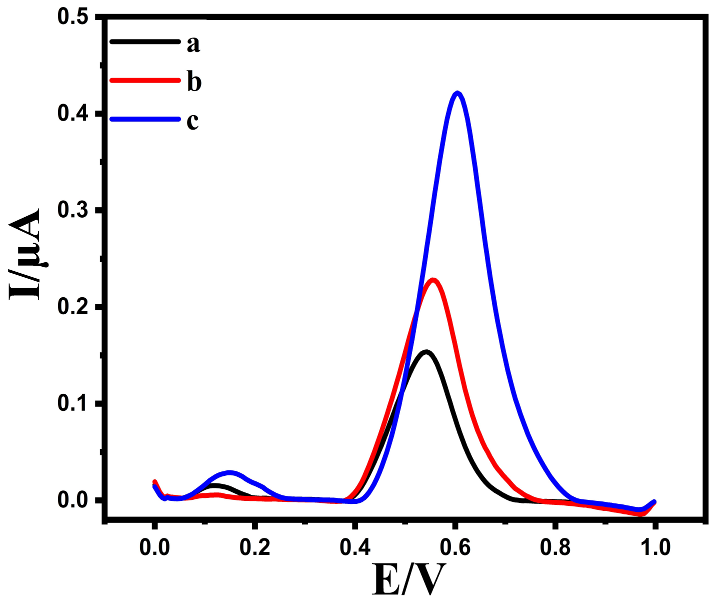

Figure S3. DPVs of the Au@$NiFe_2O_4$/SPE, the $NiFe_2O_4$/SPE and the bare electrode in the presence of 0.5 µM EPR containing a 0.1 M BR buffer.

$$I_p = 2.69 \text{ x } 10^5 \; ACn^{3/2}D^{1/2}v^{1/2} \tag{S1}$$

where I_p is the anodic or cathodic peak current (A), A is the electroactive area (cm^2), C is the molar concentration of the redox substance, n is the number of the transferred electron in the redox reaction, D is the diffusion coefficient of redox probe molecule (cm^2 s^{-1}) and v represents the scan rate (V s^{-1}).

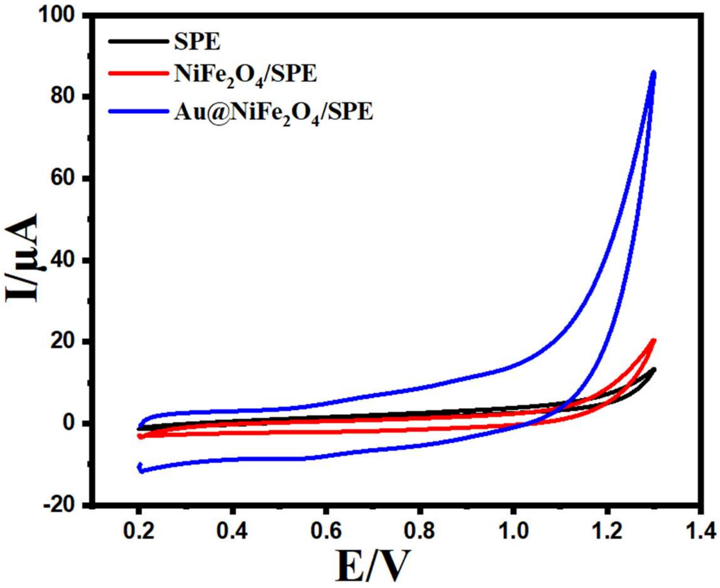

Figure S4. The CVs of the bare electrode, the $NiFe_2O_4$/SPE and the Au@$NiFe_2O_4$/SPE in the absence of EPR in B-R at pH 4.0.

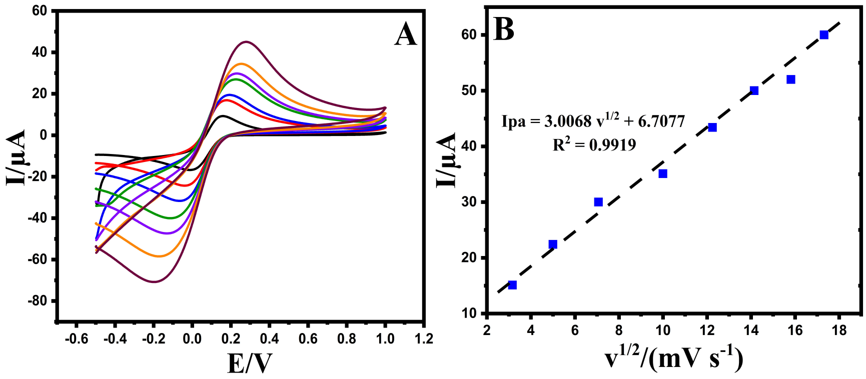

Figure S5. (**A**) CVs of the Au@NiFe2O4/SPE in the presence of 5.0 mM [Fe(CN)6]3−/4− containing 0.1 M KCl at various scan rates from 10 to 400 mV/s, (**B**) The plot of the corresponding peak current against the square root of the scan rate.

$$K^{\circ} = \frac{RT}{F^2 R_{ct} A C} \tag{S2}$$

where T is the thermodynamic temperature (298.15 K), R is the global gas constant (8.314 J K−1 mol^{-1}), F is the Faraday constant (96,485C mol−1), Rct indicates electron transfer resistance (Ω), A is the electrode surface area (cm^2), C is the $[Fe(CN)_6]^{3-/4-}$ solution concentration (5.0 mM) and K^0 represents the rate constant of a standard heterogeneous electron transfer (cm s^{-1}).

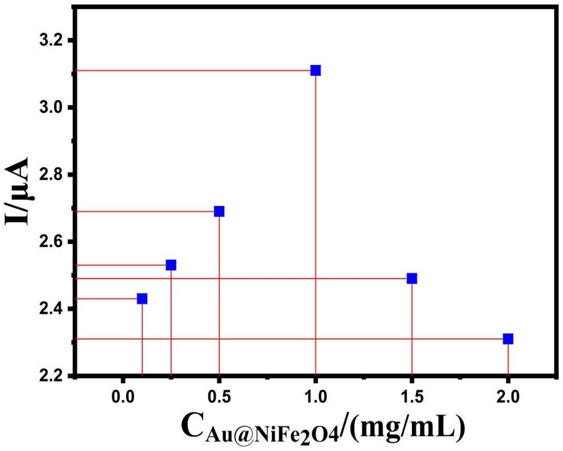

Figure S6. Effect of Au@NiFe_2O_4 concentration on EPR response using the Au@NiFe_2O_4/SPE with 5.0 μM EPR in the presence of 0.1 BR buffer at pH 4.0.

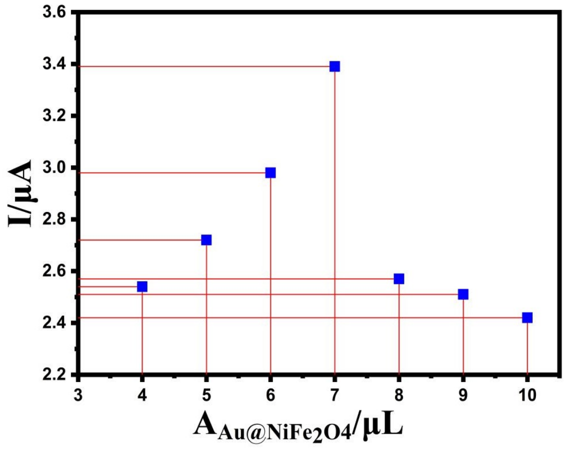

Figure S7. Effect of Au@NiFe_2O_4 amount on EPR response using the Au@NiFe_2O_4/SPE with 5.0 μM EPR in the presence of 0.1 BR buffer at pH 4.0.

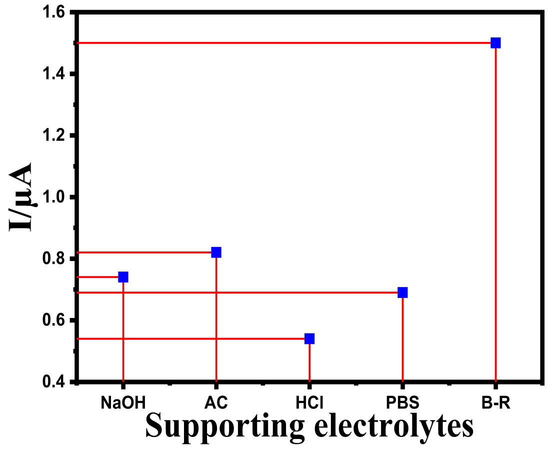

Figure S8. Effect of the Au@NiFe_2O_4/SPE in the presence of 1.0 μM EPR with various supporting electrolytes.

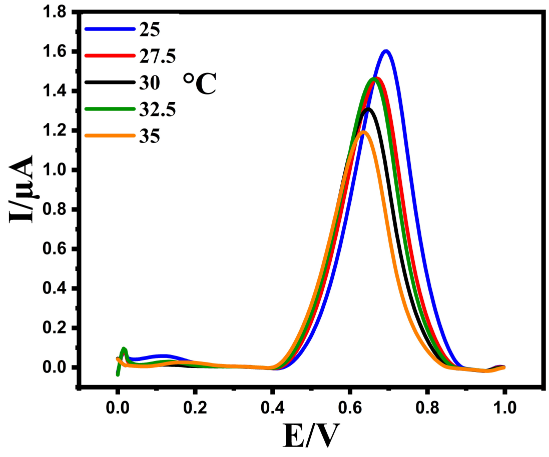

Figure S9. Effect of solution temperature on EPR response using the Au@$NiFe_2O_4$/SPE and the DPV method with 1.0 μM EPR in the presence of a 0.1 BR buffer at pH 4.0.

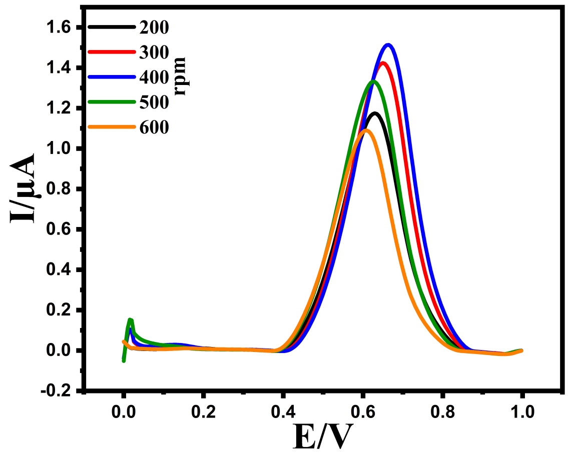

Figure S10. Effect of solution stirring rates on EPR response using the Au@$NiFe_2O_4$/SPE and the DPV method with 1.0 μM EPR in the presence of a 0.1 BR buffer at pH 4.0.

Laviron's theory:

$$Epa = E^{\circ} + \left(\frac{RT}{\alpha nF}\right) \ln\left(\frac{RTk^{\circ}}{\alpha nF}\right) + \left(\frac{RT}{\alpha nF}\right) lnv \quad \text{(S3)}$$

where E° is the formal redox potential, α is the charge transfer coefficient, n is the number of electrons transferred, k° is the standard heterogeneous rate constant, v is the scan rate and the other symbols have their usual meanings. The value of αn is calculated as 0.77 from the slope of Epa vs. ln v. Generally, α is assumed to be 0.5 for a reversible electrode process.

Cottrell's equation :

$$\mathrm{I} = \mathrm{nFACD}^{\frac{1}{2}} \pi^{\frac{-1}{2}} \mathrm{t}^{\frac{-1}{2}} \quad \text{(S4)}$$

where D (cm2 s−1) shows the analyte diffusion coefficient; the analyte bulk concentrations (mol cm−3) are shown by C; F is referred to as the Faraday constant, which equals 96,485 CM; A represents the geometric areas of the electrodes (0.2 cm2); and n is the number of transferred electrons.

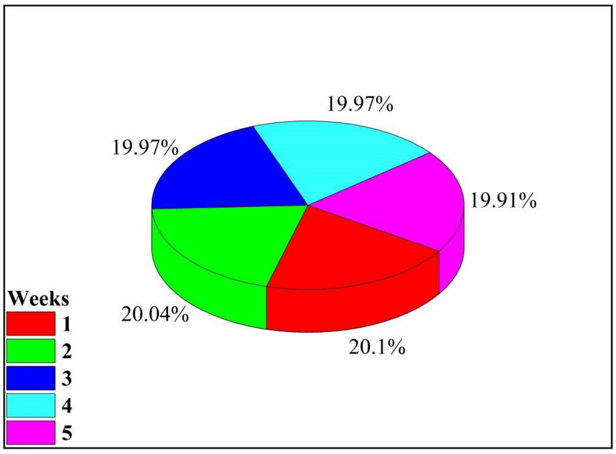

Figure S11. Long-term response stability of the Au@$NiFe_2O_4$ electrode exposed to 0.5 μM EPR over five weeks.
